# Health Challenges After a Ban on Women Working in Non-governmental Organizations in Afghanistan

**DOI:** 10.7759/cureus.41137

**Published:** 2023-06-29

**Authors:** Hosain Barati, Mirwais Ramozi, Sayed H Mousavi, Yudai Kaneda, Akihiko Ozaki

**Affiliations:** 1 Faculty of Medicine, Kateb University, Kabul, AFG; 2 School of Medicine, Hokkaido University, Sapporo, JPN; 3 Department of Breast and Thyroid Surgery, Jyoban Hospital of Tokiwa Foundation, Iwaki, JPN

**Keywords:** non-governmental organizations, global healthcare systems, afghanistan, ngos, women's rights, taliban

## Abstract

The Taliban's prohibition of women working in non-governmental organizations (NGOs) has severely impacted essential health services in Afghanistan since its enactment in late 2022. This ban has led to the suspension of numerous health and nutrition teams, reduced counseling services, and closure of safe spaces for women and girls. The significant contribution of women in the healthcare sector is evidenced by their presence in various NGOs, with their absence causing a critical shortage of female health personnel. This has further exacerbated the hunger crisis in Afghanistan, putting millions at risk. The international community urgently needs to address this issue to protect human rights and safeguard the well-being of the affected population.

## Editorial

Since December 24, 2022, the Taliban's decision to prohibit women from working in non-governmental organizations (NGOs) in Afghanistan has caused significant concern, as it impedes critical services like mother and child health care, delivery services, and health facilities [1]. The World Health Organization (WHO) states that three partners have suspended their activities in Kandahar province, affecting 23 mobile health teams and five mobile health and nutrition teams [2]. Additionally, 47% of payments go to the mahram of female staff. The United Nations International Children's Emergency Fund (UNICEF) has seen a 23% decrease in counseling services, and 77 out of 116 supported safe spaces for women and girls have been suspended in 19 provinces [3].

The decree poses challenges as women play a pivotal role in NGOs, participating in medical services, community-based activities, nutrition, and psychological support. Afghan women have significantly contributed to improving health care, with over 51% of the medical staff in Médecins Sans Frontières and over 90% of health personnel in Khost Maternity Hospital being women. In 2022, more than 3,000 of the 8,000 people employed by the International Rescue Committee in Afghanistan were women, and 30% of the 55,000 Afghan nationals working for NGOs are women [3]. However, the decree has led to an extreme shortage of female health personnel, a 20% drop in severe acute malnutrition cases screened and admitted for treatment, and a decrease in female doctors and caretakers, particularly gynecologists in remote areas [3]. Additionally, international aid agencies have suspended or decreased their activities in Afghanistan, with one-third of women-led NGOs suspending 70% of their operations [4].

Consequently, restrictions on women's participation not only exert a substantial impact on the economy but also fabricate additional obstacles to life-saving initiatives. Indeed, the current situation in Afghanistan is such that over 20 million individuals face acute hunger, with six million on the verge of famine, and five million malnourished children under the age of five and mothers [5]. The Ministry of Public Health in Afghanistan stated that female NGO workers in the health sector are exempt from the decree, but the statement's meaning is unclear, leaving organizations concerned for their female staff's safety. Therefore, the urgent need for national and international awareness about the implications of such decrees against women's rights, which result in long-term socioeconomic difficulties, particularly in the health sector, is imperative.

Conclusion

In conclusion, given the detrimental consequences of such decrees against women's rights, which will result in long-term socioeconomic complications, particularly in the health sector, we call for global support and awareness on this issue so that female NGO staff members can keep playing a vital role in transportation services, community-based activities, nutrition, and psychological support for the Afghanistan population.
